# Association of *BsmI* variant of vitamin D receptor gene with polycystic ovary syndrome: A case-control study

**DOI:** 10.18502/ijrm.v13i10.7772

**Published:** 2020-10-13

**Authors:** Nasim Ramezani, Maryam Ostadsharif, Hashem Nayeri

**Affiliations:** ^1^Department of Biochemistry, Falavarjan Branch, Islamic Azad University, Isfahan, Iran.; ^2^Department of Medical Basic Sciences, Isfahan (Khorasgan) Branch, Islamic Azad University, Isfahan, Iran.; ^3^Department of Medical Biotechnology, Isfahan (Khorasgan) Branch, Islamic Azad University, Isfahan, Iran.

**Keywords:** Vitamin D receptor, Polycystic ovary syndrome, Genetic association study.

## Abstract

**Background:**

Polycystic ovarian syndrome (PCOS) is an endocrine disorder that affects women's fertility and causes alterations such as obesity, insulin resistance, menstrual irregularities, and polycystic ovaries. The results of the studies show that the issue of vitamin D and vitamin D receptor (VDR) is controversial for PCOS susceptibility.

**Objective:**

To investigate the association of *BsmI* polymorphism in the *VDR* gene with metabolic parameters in obese PCOS women.

**Materials and Methods:**

In this case-control study, 38 obese subjects with PCOS and 40 unrelated obese individuals were evaluated to determine the allelic and genotypic frequency of *BsmI* variant by Polymerase Chain Reaction Restriction Fragment Length Polymorphism method. Body Mass Index, parathyroid hormone, phosphorus, and calcium were evaluated in all participants.

**Results:**

*BsmI* (rs1544410), (A/G) AA, AG, GG, A, and G percentage of genotypic/allelic frequencies were 65.8, 26.3, 7.9, 78.9, and 21.1 in cases and 57.5, 40, 2.5, 77.5, and 22.5 in controls, respectively. Statistical analysis revealed that the differences in genotypic (p = 0.31)/allelic (p = 0.83) frequencies and dominant (p = 0.45)/recessive (p = 0.35) models between the cases and controls were not significant. This study indicates no association between the *BsmI* genotypes and metabolic parameters.

**Conclusion:**

It can be concluded that VDR *BsmI* (rs1544410) Intron 8 (A > G) was not associated with obesity along with PCOS susceptibility in the studied groups.

## 1. Introduction

Vitamin D is present in many biological processes and takes a leading role in hormonal and metabolic disorders related to female reproductive system such as Polycystic Ovarian Syndrome (PCOS), which is considered as the most prevalent female endocrine disorder (1, 2). Alterations in insulin pathway, sex hormone production, calcium, phosphorus, and parathyroid hormone (PTH) hemostasis presented in PCOS are also shown to be affected by vitamin D (3-5). The processes brought about vitamin D are mediated via the vitamin D receptor (VDR), a nuclear receptor with a DNA-binding domain that acts through vitamin D response elements (6, 7). In the *VDR* gene, a number of allelic variations have been described, including restriction fragment length polymorphisms -which have an association with some of the patterns presented by PCOS- such as *Fok*I (C/T) (rs10735810/rs2228570), *BsmI* (A/G) (rs1544410), *Apa*I (A/C) (rs7975232), *Tru*9I (G/A) (rs757343), and *Taq*I (T/C) (rs731236). The *BsmI* SNP is located in intron 8 near the 3'end of the *VDR* gene. It may affect the VDR translation activity or mRNA stability (8).

The finding of Mahmoudi and colleagues demonstrated a significant association between VDR *BsmI* “Bb” genotype and insulin resistance in women with PCOS (9). The result of a study in south India also illustrated the association between *BsmI* A/G (rs1544410) and PCOS risk in women (10). However, in other studies such as the one conducted on Iranian Azeri Turkish women, no significant differences were observed in the genotypic/allelic frequencies between the cases and controls regarding *BsmI* (rs1544410) (11). Vitamin D plays an important role in regulating calcium-phosphorus homeostasis. Irregularity in calcium balance may lead to disruption of follicular development in women and hence affect the pathogenesis of PCOS (12). Studies show that vitamin D deficiency can be a cause of metabolic syndrome and insulin resistance in PCOS, however, whether vitamin D is linked to endocrine and fertility parameters in subjects with PCOS is unclear (13, 14).

Therefore, due to the effect of serum levels of PTH and phosphorus on PCOS, as well as the role of calcium in oocyte maturation and early embryonic development, the present study was designed to investigate the possible associations among the *BsmI* variant of *VDR* gene and PTH, phosphorus, and calcium of obese women with PCOS in a sample of Iranian population.

## 2. Materials and Methods

### Design and participants

This case-control study was conducted at the Isfahan Fertility and Infertility Center (IVF center), Isfahan, Iran, during May-September 2016; 38 obese women with PCOS as the case group and 40 obese women without PCOS as a control group, both aged 18-40 yr, and Body Mass Index (BMI) > 30 were enrolled in the study. The presence of menstrual disorders such as oligomenorrhea (six or fewer menses per year), amenorrhea (no menses in the last six months), hyper-androgenism (clinical-biochemical signs) like hirsutism (Ferriman-Gallwey score ≥ 6), acne, or alopecia and elevated androgen levels (testosterone normal range < 0.77 ng/ml and free testosterone normal range < 3.18 pg/ml) confirmed the diagnosis of PCOS. While the patient group included obese women with BMI ≥ 30, had PCOS, were married and infertile, and had no familial history of PCOS, the control group included healthy obese women with BMI ≥ 30, married, with or without children and also without a family history of PCOS. However, women diagnosed with PCOS and those from the control group who had been on medications known to affect the endocrinal system, carbohydrate, lipid, and calcium metabolism for more than three months prior to the initiation of the study were excluded.

### Biochemical measurements

Blood samples were collected from the control group between days 2 and 6 of a menstrual cycle or during a spontaneous bleeding episode and progestin-induced menstrual cycle in patients with PCOS. Overnight fasting blood samples were collected and instantly centrifuged. Next, sera were isolated and frozen at -20°C until analyzed. PTH Intact AccuBind ELISA Kits (#9025-300, Monobind Inc.USA) was used to measure the concentration of PTH in serum samples from all 78 participants. Serum level of calcium and phosphorus was quantified with Calcium Colorimetric Assay Kit (#K380-250, BioVision Inc, USA) and Phosphorus (Pi) Colorimetric Assay Kit (#E-BC-K245-S, Elabscience, China), respectively.

**Figure 1:**
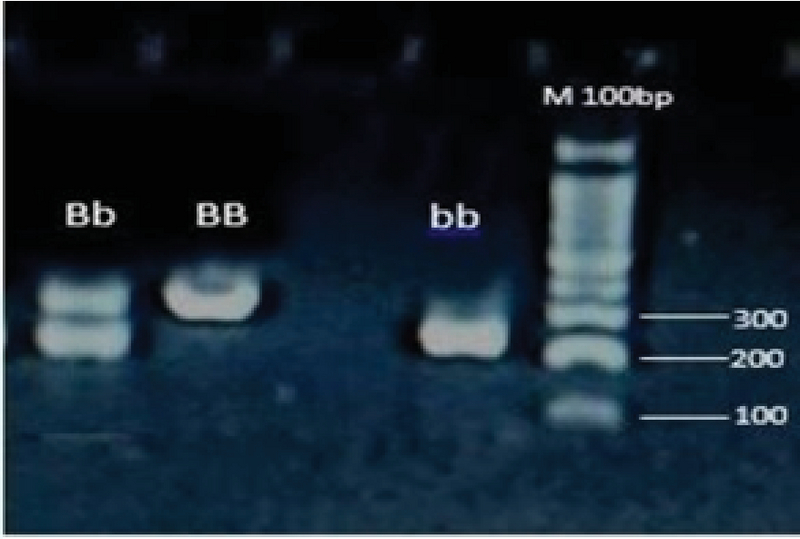
Restriction pattern of *VDR* gene fragment with *BsmI*, M: Marker.

For DNA analysis, blood samples were collected in tubes containing EDTA and stored at 4°C. Genomic DNA was purified from whole blood using a commercial isolation kit (Iraizol#1004, RNA Biotechnology Company, Iran). Genotypes of VDR *BsmI* in intron 8 A/G (rs1544410) were determined using the Polymerase Chain Reaction Restriction Fragment Length Polymorphism (PCR-RFLP) method in subjects with/without PCOS. The optimized primer pair sequences were 5'-GGAGACACAGATAAGGAAATAC-3' as *BsmI*-F and 5'-CCGCAAGAAACCTCAAATAACA-3' as *BsmI*-R. The PCR amplification was carried out in a BIO RAD system (T100TM Thermal Cycler). Twenty ml of the PCR reaction system consisted of 10 µl Taq 2X Master Mix with 1.5 mM MgCl2 (# A180306, AMPLIQON, Denmark) 10 pmol of each *BsmI*-F and *BsmI*-Rprimers and 10 ng genomic DNA. PCR was performed with an initial denaturation at 96°C for 5 min, followed by 35 cycles of denaturation at 94°C for 45 sec, annealing at 60°C for 45 sec, and extension at 72°C for 45 sec. The final extension was at 72°C for 5 min. PCR products were separated in 2% agarose gel. The length of PCR product was 248 bp. Enzymatic digestion was performed by the *BsmI* (#R0134S, New England Biolabs) enzyme in accordance with the manufacturer's instructions. The *BsmI* polymorphic site revealed two possible alleles, allele B/A (absence of a restriction site) and b/G (presence of a restriction site). The genotypes were defined as BB (one band at 248 bp), bb (two bands at 73 and 175 bp), or Bb (three bands at 248, 73, and 175 bp) (Figure 1).

### Ethical consideration

The study was approved by the Research Committee of Islamic Azad University, Falavarjan Branch (Code: 17230520942004). Informed consent was obtained from all participants.

### Statistical analysis

Chi-square test (χ2) was used to compare the distribution of the allele and genotype frequencies and determination of Hardy-Weinberg equilibrium for *BsmI* polymorphism. The χ2 value was calculated using the Statistical Package for the Social Sciences, version 20.0, SPSS Inc, Chicago, Illinois, USA (SPSS). Additionally, independent t test and Man-Whitney test were used to detect the differences between the cases and controls in respect to their characteristics (p < 0.05), and the result was statistically significant.

## 3. Results

Table I summarizes the characteristics of the subjects with and without PCOS. In our study, the mean age of the 38 participants with PCOS was 28.58 ± 5.83 yr, and the mean age of 40 controls was 31.34 ± 5.5 yr (p < 0.001). There were no significant differences between women with PCOS and controls regarding clinical characteristics such as BMI (p = 0.072), serum level of PTH (p = 0.32), serum level of calcium (p = 0.79), and phosphorus (p = 0.61).

Table II demonstrates the allelic and genotypic frequencies of VDR *BsmI* polymorphism in intron 8 (A/G) (rs1544410). VDR *BsmI* genotypic distribution in controls were in Hardy-Weinberg equilibrium (χ2 = 0.864, p = 0.52). The B and b alleles' frequencies of VDR *BsmI* polymorphism were 78.9% and 21.1% in cases and 77.5% and 22.5% in controls regarding the “A” and “G” alleles. Also, the frequencies of BB, Bb, and bb genotypes of the *BsmI* variant were 65.8%, 26.3%, and 7.9% in cases and 57.5%, 40%, and 2.5% in controls, respectively. There were no significant differences in genotypes (p = 0.31) and allele (p = 0.83) frequencies between the obese PCOS and obese healthy controls. According to result of the Fisher's exact test and χ2, recessive (p = 0.35) and dominant (p = 0.45) genotypes did not show any significant association with the disease.

Table III presents the distribution of serum level of PTH, calcium, and phosphorus according to the genotypes observed in individuals with and without PCOS. Because of the limited frequencies of genotype bb in the VDR *BsmI* polymorphism, we combined Bb and bb genotypes as a group. Control women with BB genotype were found to have a relatively higher mean than those with Bb + bb genotype. However, none of the biochemical traits showed significant differences with respect to the *BsmI* genotypes (Table III). Although, the level of serum PTH was significantly different between women with BB genotype and bb + Bb among control group (p = 0.04), the level of PTH showed no significant differences among women with PCOS with BB or Bb + bb genotypes (p = 0.88) (Table III). The level of phosphorus and calcium in women with different genotype BB and Bb + bb in both case and health control groups demonstrated no significant elevation.

**Table 1:** The clinical characteristics of PCOS women


	**Cases**	**Controls**	*P*-value
**Number of subjects**	38	40	-
**Age (Yr)**	28.58 ± 5.83	31.34 ± 5.5	< 0.001${}^{a}$
**BMI (kg/m${}^{2}$)**	33.82 ± 3.39	32.39 ± 3.32	0.07${}^{a}$
**Serum level of PTH (pg/ml)**	31.36 ± 12.67	19.42 ± 10.07	0.32${}^{b}$
**Serum level of calcium (mg/dl)**	9.61 ± 0.48	9.59 ± 0.55	0.79${}^{b}$
**Serum level of phosphorus (mg/dl)**	3.35 ± 0.4	3.49 ± 0.44	0.61${}^{b}$
BMI: Body mass index; PTH: Parathyroid hormone, ${}^{a}$Independent sample *t* test and ${}^{b}$Mann-Whitney test			

**Table 2:** Allelic and genotypic frequencies of VDR *BsmI* in cases and controls


	**Controls**	**Cases**	*P*-value
	**Number (%)**	**Number (%)**	
**Allele**			
**B (A)**	62 (77.5)	60 (78.9)	0.83${}^{a}$
**b (G)**	18 (22.5)	16 (21.1)		
**Total**	80 (100)	76 (100)	
**Genotype**			
**BB (AA)**	23 (57.5)	25 (65.8)	0.31${}^{b}$
**Bb (AG)**	16 (40.0)	10 (26.3)	
**Bb (GG)**	1 (2.5)	3 (7.9)		0.35${}^{b}$
**Total**	40 (100)	38 (100)		
**Recessive**				
**BB + Bb**	39 (97.5)	35 (92.1)
**bb**	1 (2.5)	3 (7.9)	
**Total**	40 (100)	38 (100)
**Dominant**			
**BB**	23 (57.5)	25 (65.8)	
**Bb + bb**	17 (42.5)	13 (34.2)	
**Total**	40 (100)	38 (100)
${}^{a}$Chi-square test and ${}^{b}$Fisher's exact test			

**Table 3:** *BsmI* polymorphism and PTH (pg/ml), Ca (mg/dl), P (mg/dl)


	**Bb + bb**	**BB**	*P*-value${}^{a}$
**Cases**				
	**PTH**	26.76 ± 13.89	32.76 ± 17.95	0.88
	**Ca**	9.54 ± 0.53	9.64 ± 0.46	0.57
	**P**	3.45 ± 0.46	3.58 ± 0.37	0.31
**Controls**				
	**PTH**	26.98 ± 14.99	13.83 ± 8.91	0.04
	**Ca**	9.49 ± 0.45	9.67 ± 0.61	0.43
	**P**	3.49 ± 0.56	3.48 ± 0.35	0.67
PTH: Parathyroid hormone; Ca: Calcium; P: Phosphorus; ${}^{a}$ Mann-Whitney test				

## 4. Discussion

In this study, we examined the *BsmI* polymorphism among obese women with and without PCOS as cases and controls, respectively, and tested for their association with PTH, Ca, and P phenotypes. *BsmI* polymorphism in the *VDR* gene is located on the 3' regulatory region. The three adjacent RFLP for *BsmI*, *Apa*I, and *Taq*I, which are located near the 3' end of the gene is known to be involved in regulating expression, particularly through the control of mRNA stability (15). Therefore, the frequency of *BsmI* genotypes and alleles seem to play a role in the disease. Although, in our study, there was no significant difference in B and b alleles and genotypes between the control and patient groups, the results of other populations were different. In our population, BB genotype was more frequent in both groups than other genotypes. However, there was no significant difference in the dominant and recessive models in the studied groups.

The results of studies on *VDR* gene polymorphisms in other populations showed an association of VDR *BsmI* A/G, *Apa*I A/C, and *Taq*I T/C SNPs with PCOS risk in South Indian women (10). Moreover, the result of a study by Mahmoudi and colleagues in 35 Iranian women with PCOS in 2015 determined an association between the *VDR* gene *BsmI* and *Apa*I polymorphisms and PCOS risk (9). Another study, conducted by Mahmoudi, focused on finding an association between the *Fok*I, *BsmI*, *Apa*I, *Taq*I polymorphisms and PCOS. This study selected 162 patients aged 19-42 yr, diagnosed according to the National Institute of Child Health and Human Development criteria. In his research, he reported that the *VDR* gene *Apa*I polymorphism is associated with PCOS (1). Bagheri and colleagues studied the *Fok*I and *BsmI* variations of the *VDR* gene in genetic susceptibility to PCOS in 46 Iranian, Azeri Turkish women. Their results showed no significant differences with PCOS susceptibility in the studied group (11). Further, Ranjzad and colleagues investigated a possible relation between the *Fok*I, *BsmI*, *Apa*I, *Taq*I, and *Tru*9I polymorphisms with biochemical and metabolic parameters in 56 Iranian PCOS women. Results revealed significant associations between decreased levels of sex hormone binding globulin (SHBG) and both VDR *BsmI* “GG” (p = 0.009) and adiponectin (ADIPOQ) *BsmI* “CC” (p = 0.016) genotypes, suggesting that the “G” allele in homozygous is a risk factor for PCOS (16).

Additionally, the results of a meta-analysis study by Han and co-workers suggested that VDR *BsmI* variant G allele might be a susceptibility marker of metabolic syndrome and VDR *Taq*I variant C allele might be a susceptibility marker of PCOS (17). Wehr and co-authors carried out a cohort study including 545 women from Austria with PCOS in order to investigate the association between VDR polymorphisms and PCOS susceptibility. Their research results revealed no association of VDR *BsmI*, *Fok*I, and *Taq*I polymorphisms with anthropometric, endocrine, or metabolic parameters (18).

Also, the results obtained by Jedrzejul and colleagues were suggestive of a lack of association among the homogeneous classic PCOS phenotype, vitamin D deficiency, and *VDR* gene polymorphisms in lower Silesian women (19). Of course, several research/studies have been performed on the relationship between *VDR* gene polymorphisms and PCOS disease in various populations, which showed some association beside some lack of association (20-23). In accordance with our study, Kumar and co-workers showed that VDR *BsmI* (rs1544410) intron 8 (A/G) were not associated with PCOS susceptibility in Indian women population (24). In another study on the same population as studied by us, it was found that *Taq*I polymorphism was not associated with the risk of PCOS (25). The results of various studies indicated that the relationship between VDR gene polymorphisms and PCOS among different ethnicities was controversial (4). On the other hand, in the Iranian population, studies have been conducted on the association of microRNAs (miRNA) that indicate the association with PCOS (26).

Since vitamin D and its metabolites are responsible for gene expression containing vitamin D response elements including PTH (27), there seems to be an association between the *VDR* gene variants and PTH secretion. However, in our study, there was no significant association between the *BsmI* genotypes and serum PTH levels. Findings of Zˇofkova and colleagues showed that FokI polymorphism of the *VDR *gene is closely related to the magnitude of PTH secretion and/or degradation in postmenopausal women (28). Although studies have reported that women with PCOS (29, 30) had a higher level of PTH and phosphorus, in our study, obese women with Bb + bb genotype in control group had higher serum levels than obese women with BB genotype. There was also no significant difference in serum calcium and phosphorus levels between the control and patient groups in any of the genotypic groupings.

Nevertheless, the present study had several limitations. For example, its sample size and our lack of information on other VDR polymorphisms, which could have affected the results obtained in the study.

## 5. Conclusion

This study revealed that *BsmI* (rs1544410) in intron 8 of *VDR* gene has no association with obese PCOS patients in our population.

##  Conflict of Interest 

The authors declare that there is no conflict of interest.
